# Use of Mass Spectrometry to Profile Peptides in Whey Protein Isolate Medium Fermented by *Lactobacillus helveticus* LH-2 and *Lactobacillus acidophilus* La-5

**DOI:** 10.3389/fnut.2019.00152

**Published:** 2019-10-15

**Authors:** Eman Ali, Søren D. Nielsen, Salah Abd-El Aal, Ahlam El-Leboudy, Ebeed Saleh, Gisèle LaPointe

**Affiliations:** ^1^Food Hygiene Department, Faculty of Veterinary Medicine, Damanhour University, Damanhour, Egypt; ^2^Canadian Research Institute for Food Safety, University of Guelph, Guelph, ON, Canada; ^3^Department of Food Science, Faculty of Science and Technology, Aarhus University, Aarhus, Denmark; ^4^Food Control Department, Faculty of Veterinary Medicine, Zagazig University, Zagazig, Egypt; ^5^Food Hygiene Department, Faculty of Veterinary Medicine, Alexandria University, Alexandria, Egypt

**Keywords:** bioactive peptides, probiotic, whey, *Salmonella*, virulence, gene expression

## Abstract

Peptides in the 3-kDa ultrafiltrate of fermented whey protein isolate (WPI) medium could be responsible for the antivirulence activity of *Lactobacillus helveticus* LH-2 and *Lactobacillus acidophilus* La-5 against *Salmonella* Typhimurium. Non-fermented and fermented media containing 5.6% WPI were fractionated at a 3 kDa cut-off and the filtrate was analyzed by mass spectrometry. The non-fermented WPI medium contained 109 milk derived peptides, which originated from β-casein (52), αs1-casein (22), αs2-casein (10), κ-casein (8), and β-lactoglobulin (17). Most of these peptides were not found in the fermented media, except for 14 peptides from β-casein and one peptide from α_s2_-casein. Database searches confirmed that 39 out of the 109 peptides had established physiological functions, including angiotensin-converting-enzyme (ACE) inhibitory, antioxidant, antimicrobial, or immunomodulating activity. A total of 75 peptides were found in the LH-2 cell free spent medium (CFSM): 54 from β-casein, 14 from k-casein, 4 from β-lactoglobulin and 3 from αs2-casein. From these peptides, 19 have previously been associated with several categories of bioactivity. For La-5 CFSM, a total of 15 peptides were sequenced: 8 from β-casein, 5 from αs1-casein, 2 from β-lactoglobulin. Only 5 of these have previously been reported as having bioactivity. Many of the peptides remaining in the fermented medium would contain low-affinity residues for oligopeptide binding proteins and higher resistance to peptidase hydrolysis. These properties of the sequenced peptides could explain their accumulation after fermentation despite the active proteolytic enzymes of LH-2 and La-5 strains. Down-regulated expression of *hilA* and *ssrB* genes in *S*. Typhimurium was observed in the presence of La-5 and LH-2 CFSM. Downregulation was not observed for the *Salmonella oppA* mutant strain exposed to the same CFSM used to treat the *S*. Typhimurium DT104 wild-type strain. This result suggests the importance of peptide transport by *S*. Typhimurium for down regulation of virulence genes in *Salmonella*.

## Introduction

*Salmonella enterica* subsp. *enterica* serovar Typhimurium is considered a major foodborne pathogen with public health and economic concerns. This foodborne pathogen has developed resistance against a broad range of antibiotics (1). Alternative approaches to control this pathogen depending on the inhibition of virulence gene expression and stimulation of the host immune system have been suggested. These approaches cause less stress to bacterial cells to avoid the development of resistant clone (2). A Type III Secretion System (TTSS) is used by *S*. Typhimurium to inject and translocate effector proteins into host cells for adherence, attachment and invasion (3). *Salmonella* pathogenicity island I (SPI1) controls the production and activity of the Type III secretion system which enables the membrane ruffling process, in which the epithelial cell cytoskeleton is rearranged to engulf the *Salmonella* into cytoplasmic vacuoles (4). The gene *hilA* directly controls and activates all the genes of SPI1 for invasion (5). The gene *ssrB* is the main regulator of SPI2, which is responsible for systemic infection and replication of *S*. Typhimurium inside macrophages and epithelial cells (6). Down regulation of these virulence genes would be an alternative way to reduce the severity of *Salmonella* infection.

Milk proteins are a major source of bioactive peptides (7). Milk derived peptides may have effects on the digestive, immune, cardiovascular and nervous systems (8). Previous studies have suggested that bioactive metabolites produced by *L. helveticus* LH-2 and *L. acidophilus* La-5 could down-regulate virulence gene expression in both *Salmonella* and *E. coli* O157:H7 after growth in chemically defined media and milk (9–12).

Bioactive peptide production in lactic acid bacteria (LAB) is the result of the balance between proteolytic activity and peptide consumption (13). The proteolytic system of LAB consists of proteinases and peptidases which initially cleave milk proteins to oligopeptides, specific transport proteins which transport smaller peptides and amino acids across the cytoplasmic membrane and intracellular peptidases, which further degrade these peptides to small peptides and amino acids (14). The considerable amino acid auxotrophy of *L*. *helveticus* and *L. acidophilus* is consistent with the presence of many peptidases/proteases and related transport systems for amino acids and peptides (15).

The oligopeptide-binding protein (OppA) is one of the most abundant periplasmic proteins in *S*. Typhimurium*. oppA* gene expression in *S*. Typhimurium is modulated by nitrogen compounds and regulates the expression of other genes such as Lrp (leucine-responsive regulatory protein) or CodY (16). Mutation in OppA in *S*. Typhimurium leads to losing the ability to transport muropeptides and detect the nutritional or signaling peptides (17).

In this study, the proteolytic and peptidolytic activities of LH-2 and La-5 strains in Whey Protein Isolate (WPI) are explored through genome sequencing and biochemical assays on substrates. Peptide profile analysis by mass spectrometry was conducted to understand the potential factors that lead to accumulation of antivirulence peptides in the growth media. Furthermore, the correlation between *oppA* gene expression and virulence gene expression in *Salmonella* in the presence of antivirulence peptides was investigated using a *S*. Typhimurium *oppA* mutant strain.

## Materials and Methods

### Bacterial Strains and Growth Conditions

All bacterial strains used in this study were obtained from the Canadian Research Institute for Food Safety (CRIFS) except for the *Salmonella* Typhimurium *oppA* mutant, which was provided by Prof. Eric Brown, McMaster University. *Lactobacillus helveticus* LH-2 and *Lactobacillus acidophilus* La-5 probiotic strains were grown under anaerobic conditions at 37°C for 48 h on De Man, Rogosa, Sharpe broth (MRS; Thermo Scientific™ Oxoid™ Ottawa, ON, Canada). *Salmonella* Typhimurium *hilA::lux* and *Salmonella* Typhimurium *ssrB::lux* were grown on Luria-Bertani broth (LB; Life Technologies, Burlington, ON, Canada) supplemented with 50 μg/ml of ampicillin (Amp). Both constructs were grown aerobically overnight on a shaking incubator at 37°C. *S*. Typhimurium DT104 and *S*. Typhimurium *oppA* mutant strains were grown under the same condition as *S*. Typhimurium constructs, but without Amp. Solid media preparation for all strains were carried out under the same conditions but with addition of 15 g/l of agar and incubated under the same conditions.

### Preparation of *L. helveticus* LH-2 and *L. acidophilus* La-5 Cell Free Spent Medium (CFSM)

Whey Protein Isolate (WPI) (NZWPI 895, Caldic, Fonterra, USA) was dissolved at 5.6% (v/v) in sterile sugar solution. Glucose or sucrose (Fisher Scientific, Ottawa, ON, Canada) at 0.5% (v/v) were added for *L. helveticus* LH-2 and *L. acidophilus* La-5, respectively. The media were filter sterilized through 0.45 μm pore-size filters (Corning, NY 14831, Germany). An overnight culture of each strain in MRS broth was washed and inoculated at 5% (v/v) into WPI based media and incubated anaerobically at 37°C for 48 h. Following growth, bacterial cells were removed by centrifugation at 15,000 × *g* for 30 min at 4°C. The supernatant was filtered through a 0.20 μm pore-size filter (Corning) to obtain sterile CFSM which was subsequently freeze-dried (VirTis, Genesis, USA) for 72 h and stored at −80°C.

### Effect of CFSM on Gene Expression in *S*. typhimurium Reporter Constructs

For initial screening of the antivirulence effects of CFSM, bioluminescent reporter strains *S*. Typhimurium *hilA::lux* and *ssrB::lux* (18) were used. Briefly, the *lux*CDABE operon from *Xenorhabdus luminescens* was isolated and cloned with an Amp resistance gene into a plasmid (pSB377), which was further fused with the *hilA* and *ssrB* promoter regions, separately. The expression of the *lux* genes is controlled by these promoter regions, so that light is emitted when the gene is expressed. Each overnight culture of the constructs was diluted 1:100 with fresh LB broth with and without supplementation of 10% (v/v) of neutralized (pH 7) CFSM from *L. helveticus* LH-2 and *L. acidophilus* La-5, which had been reconstituted in sterile deionized water at 10-fold concentration compared to the initial medium. The samples were incubated aerobically at 37°C with shaking for 3 h. Luminescence was measured with the FB 12 luminometer (Berthold Detection Systems, Pforzheim, Germany). The results are presented as Relative Light Units (RLU)/OD_600_ nm.

### Effect of CFSM and Mixture of Synthetic Peptides on Transcription of Virulence Genes of *S*. typhimurium by RT-qPCR

#### RNA Extraction

RNA was extracted from *S*. Typhimurium DT104 and *S*. Typhimurium *oppA* mutant following 3 h growth in fresh LB broth with and without 10% neutralized (pH 7) CFSM with shaking at 37°C, 2-ml samples were centrifugated at 5,000 × g for 4 min at RT. The supernatant was discarded and cells were mixed with 1 ml of RNA Protect reagent (Qiagen Inc., Mississauga, ON, Canada) and incubated for 5 min at RT. Cells were collected again by centrifugation at 5,000 × g for 10 min at RT. Cell pellets were suspended in 200 μl of Tris-EDTA buffer, pH 8.0 (Fisher Scientific, Ottawa, ON, Canada), 60 μl of 20 mg/ml lysozyme (Fisher Scientific) and 20 μl of proteinase K (Qiagen). The suspensions were incubated at 37°C for 1 h with shaking at 450 rpm. RNeasy Plus Mini Kit (Qiagen) was used to extract RNA from all samples following the manufacturer's instructions. DNA was eliminated by using RNase-Free DNase Set (Qiagen). In brief, 40 μl of total RNA was incubated for 10 min at RT with 2.5 μl DNase I stock solution, 10 μl RDD buffer in a total volume of 100 μl. RNA purification and concentration were performed with RNeasy MinElute Cleanup kit (Qiagen) and solubilized in 30 μl molecular-grade water. The quantity of quality RNA was determined by measuring the absorbance at 260 and 280 nm using a NanoDrop 1000 spectrophotometer (Thermoscientific, Wilmington, DE 19810, USA). RNA integrity was verified by gel electrophoresis.

The same procedures were used for a mixture of the following synthetic peptides (GLDIQKVAGT, ELNVPGEIVES, DVENLHLPLPL, GVSKVKEAMAPKH, SSSEESITRIN) (Synpeptide) at a concentration of 0.2 mg/ml for each peptide.

#### Reverse Transcription

The purified RNA was used for reverse transcription by using high-capacity cDNA reverse transcription kit (Applied Biosystems, Burlington, ON, Canada). RNA (1 μg) was mixed with 2 μl of 10× RT buffer, 2 μl of 10× random hexamer primers, 0.8 μl of 25× dNTP (100 mM), 1 μl of Multiscribe reverse transcriptase (50 U/ml) in a total volume of 20 μl. A no reverse transcription control was included to confirm the absence of contaminating DNA. The synthesis of cDNA was conducted using a Mastercycler Gradient Thermocycler (Eppendorf, Mississauga, ON, Canada) under the following settings: 25°C for 10 min, 37°C for 120 min, 85°C for 5 min and a holding step at 4°C. The cDNA was stored at −20°C until use.

#### RT-qPCR

A ViiA^TM^ 7 Real-Time PCR System (Applied Biosystems, Burlington, ON, Canada) and PowerUp™ SYBR™ Green Master Mix (Applied Biosystems) were used for RT-qPCR. The primers (Table 1) were synthesized by Eurofins Genomics (Huntsville, USA). The primer efficiency was calculated as described before (23) with the formula: *E* = 10 ^(−1/slope)^. The PCR was performed in a total volume of 20 μl; 10 μl of PowerUp™ SYBR™ Green Master Mix, 1.6 μl of forward primer (5 μM), 1.6 μl of reverse primer (5 μM), 5 μl of 1/10 diluted cDNA and 1.8 μl of molecular-grade water with the final primer concentration of 400 nM for all the genes except 16S (ribosomal RNA gene) and *rpoD* (sigma factor). For these two genes, a final primer concentration of 200 nM was used with reaction mixture, 10 μl of SYBR® Select Master Mix, 0.8 μl of forward primer (5 μM), 0.8 μl of reverse primer (5 μM), 5 μl of 1/10 diluted cDNA and 3.4 μl of molecular-grade water. Each PCR was performed in triplicate in the 96 well plates (MicroAmp™ Optical 96-Well Reaction Plate with Barcode, Fisher Scientific, Canada). PCR conditions were as follows: UDG activation at 50°C for 2 min and Dual-Lock™ DNA polymerase activation at 95°C for 2 min, followed by 40 repeated cycles of denaturation, annealing and amplification, at 95°C for 15 s, 54°C for 30 s and 72°C for 45 s. Subsequently, a default dissociation curve (95°C for 15 s, 60°C for 1 min and 95°C for 15 s) was performed in the instrument and specific amplicon was verified by a single melting-temperature peak. The transcript levels were normalized to the geometric average of expression for all housekeeping genes for each sample (24). The relative changes in gene expression were calculated by using the formula: dCT = CT (target)-CT (Normalizer), then ddCT is calculated by subtracting the dCT of the untreated sample from the treated one: ddCT = dCT (treated)- dCT (untreated). The relative gene expression is calculated as, 2^−ddCT^. Finally, a fold change was calculated as −1/2^−ddCt^ (25).

**Table 1:** Oligonucleotides used in this study.

**Gene**	**Function**	**Primer sequences**	**References**
*hilA*	Transcriptional regulator of SPI-1	F: 5′-TGTCGGAAGATAAAGAGCAT-3′R: 5′-AAGGAAGTATCGCCAATGTA-3′	(19)
*ssrB2*	Transcriptional regulator of SPI-2	F: 5′-TGGTTTACACAGCATACCAA-3′R: 5′-GGTCAATGTAACGCTTGTTT-3′	(19)
*16S*	*16S* rRNA gene, used as housekeeping gene	F: 5′-CAAGTCATCATGGCCCTTAC-3′R: 5′-CGGACTACGACGCACTTTAT-3′	(20)
*ropD*	Sigma factor, used as housekeeping gene	F: 5′-GTGAAATGGGCACTGTTGAACTG-3′R: 5′-TTCCAGCAGATAGGTAATGGCTTC-3′	(21)
*gmk*	Guanylate kinase, used as housekeeping gene	F: 5′-TTGGCAGGGAGGCGTTT-3′R: 5′-GCGCGAAGTGCCGTAGTA AT-3′	(22)

### Genome Sequencing of *L. helveticus* LH-2 and *L. acidophilus* LA-5

Genomic DNA was extracted from *L. helveticus* LH-2 and *L. acidophilus* La-5 by using the UltraClean Microbial DNA Isolation Kit (Mo-Bio Laboratories, Inc., Canada) according to the manufacturer's instructions. DNA was eluted in 10 mM Tris-HCl (pH 8.0). The concentration and the purity of the purified DNA was measured at 260 and 280 nm using a Nanodrop 1000 spectrophotometer. The integrity of the DNA was verified by agarose (1%) gel electrophoresis. Extracted DNA samples were stored at −20°C. Library preparation and sequencing were performed at the Plateforme d'Analyses Génomiques of the Institut de Biologie Intégrative et des Systèmes (IBIS, Université Laval Quebec, Canada). In short, the libraries were prepared using 500 ng of mechanically fragmented DNA by a Covaris M220 (Covaris) using the NEBNext Ultra II kit (New England Biolabs). TruSeq HT adapters (Illumina) were ligated instead of NEBNext adaptors. The libraries were checked for quality using Bioanalyzer and quantified with Picogreen. The libraries were sequenced on a fraction of a MiSeq run (v3 600 cycles, Illumina) following the manufacturer's instructions. *De novo* assembly of the reads was performed using CLC Bio Genomic Workbench version 10.1 (Qiagen Inc., Mississauga, ON, Canada) at the Genomics Facility of the Advanced Analysis Centre, University of Guelph. Both the assembled reads and the *de novo* assembled contigs were BLAST searched (https://blast.ncbi.nlm.nih.gov/Blast.cgi). Comparative genomic analysis of the proteolytic system was conducted using other related strains *L. helveticus* CNRZ 32 (abbreviation LAC LHE, accession number CP002081) for *L. helveticus* LH-2 and *L. acidophilus* NCFM (abbreviation LAC, accession number CP000033) for *L. acidophilus* La-5 using the NCBI microbial genome database and Blast alignment tools.

### Measurement of the Cell Envelope Proteinase (CEP) Activities

*L. helveticus* LH-2 and *L. acidophilus* La-5 were grown anaerobically in WPI—sugar based medium supplemented with 10 mM CaCl_2_.2H_2_O at 37°C for 24 h. The cells were harvested by centrifugation at 15,000 × g for 10 min at 4°C and washed twice with 10 mM CaCl_2_-saline solution. The supernatant designated as extracellular extract (EE) was used for the measurement of cell lysis rate while the cells were re-suspended in Tris buffer (50 mM, pH 7.8) to an optical density at 600 nm (OD_600_) of approximately 10 and were used for CEP and aminopeptidase enzyme assays.

The CEP activity assay of intact cells was performed using the chromogenic substrate N-succinyl-Ala-Ala-Pro-Phe-p-nitroanilide (Sigma, Markham, ON, Canada) as described previously (26). The reaction mixture was consisted of 200 μl resuspended cells, 287.5 μl phosphate buffer (0.2 M, pH 7.0), 225 μl 5 M NaCl and 37.5 μl N-succinyl-Ala-Ala-Pro-Phe-p-nitroanilide (20 mM). The reaction components were mixed gently and incubated at different temperatures (35, 40, 45, 50, and 55°C) for 30 min to determine the effect of temperature on the proteinase activity. The reactions were stopped by adding 175 μl of 80% (v/v) acetic acid followed by centrifugation at 13,000 × g for 5 min. The release of p-nitroanilide (pNA) was measured at 410 nm using Beckman DU 500-Spectrophotometer. The enzyme activity was calculated using a molar absorption coefficient (ε) of 8,800 mol^−1^cm^−1^. One unit of protease activity was defined as 1 nmol of p-nitroanilide released per minute. The specific protease activity was calculated as one unit of protease from 1 mg of cell protein. The protein content was estimated using the method of Bradford (27). Bovine serum albumin (Thermoscientific, Rockford, USA) was used as a standard to measure the protein content.

### Aminopeptidase Activities

Suspensions of harvested cells with OD_600_ of approximately 10 were used for preparation of intracellular extracts (IE) according to Pescuma et al. (28), cells were disrupted by using Microbead tubes (Mo-Bio Laboratories, Inc., Canada) in a microbead tube vortex for 6 min at maximum speed with 1 min interruption on ice after each minute. Beads, cell debris and unbroken cells were removed by centrifugation (10,000 × *g* at 4°C for 5 min) and the supernatant fluid was designated as the intracellular enzymatic extract (IE) which was maintained on ice and immediately used for enzymatic assays. Intracellular aminopeptidase activity was assayed with the chromogenic substrate Lys-ρNa (Sigma) as previously described (29) by incubation of 100 μl of IE with 400 μl of Tris-HCl buffer (50 mM, pH 7.0) and 50 μl Lys-ρNa (10 mM) at five temperatures (35, 40, 45, 50, and 55°C) for 20 min to determine the effect of temperature on the aminopeptidase activity. The reaction was stopped by addition of 1 mL of 30% acetic acid. The concentration of ρ-nitroanilide released was quantified at 410 nm. Enzyme activities were calculated by using a molar absorbance coefficient of 9,024 mol^−1^cm^−1^ under the assay conditions. One unit of enzyme activity was defined as the amount of enzyme required to release 1 nmol of ρ-nitroanilide per min under the assay conditions. The specific peptidase activity was calculated as one unit of peptidase from 1 mg of cell protein. Aminopeptidase activity was measured in EE by the same procedures to detect rate of cell lysis.

### Identification of Peptides Through Liquid Chromatography–Mass Spectrometry Analysis (LC–MS)

Filtered CFSM was fractionated at three successive cut-off protein concentrator sizes 100, 50, and 3 kDa, respectively, using GE Healthcare Vivaspin™ 20 protein concentrator (Fisher Scientific). The filtrate was freeze dried for 72 h. A control sample of filter sterilized WPI CFSM was included. The freeze-dried filtrate of 30 ml CFSM was reconstituted in 300 μl Milli-Q water and peptides were identified on an Agilent 1200 HPLC liquid chromatograph interfaced with an Agilent UHD 6530 Q-Tof mass spectrometer at the Mass Spectrometry Facility of the Advanced Analysis Centre, University of Guelph (30). Briefly, a C18 column (Agilent Advance Bio Peptide Map, 100 × 2.1 mm 2.7 μm) was used for chromatographic separation with the following solvents, water with 0.1% formic acid for A and acetonitrile with 0.1% formic acid for B. The mobile phase gradient was 2% B increasing to 45% B in 40 min and then to 55% B in 10 more minutes followed by column wash at 95% B and 10-min re-equilibration with a flow rate 0.2 mL/min.

The mass spectrometer electrospray capillary voltage was maintained at 4.0 kV and the drying gas temperature at 350°C with a flow rate of 13 L/min. Nebulizer pressure was 40 psi and the fragmentor was set to 150. Nitrogen was used as both nebulizing and drying gas, and collision-induced gas. The mass-to-charge ratio was scanned across the m/z range of 300–2,000 m/z in 4 GHz extended dynamic range positive-ion auto MS/MS mode. Three precursor ions per cycle were selected for fragmentation. The sample injection volume was 20 μl. Raw data files were loaded directly into PEAKS 8 software (Bioinformatics Solutions Inc.) and subjected to *de novo* sequencing with SwissProt database searching. The tolerance values used were 15 ppm for parent ions and 0.05 Da for fragment ions. See Supplementary Table S1 for information used to identify peptides.

### Statistical Analysis

All experiments were carried out three independent times with triplicates of each sample. Means and standard deviations were analyzed using ANOVA followed by Tukey's *post hoc* test with a *P*-value of ≤ 0.05 considered significant.

## Results

### Activity of *L*. *helveticus* LH-2 and *L. acidophilus* La-5 CFSM on Bioluminescent Reporter Strains

**Figure 1:**
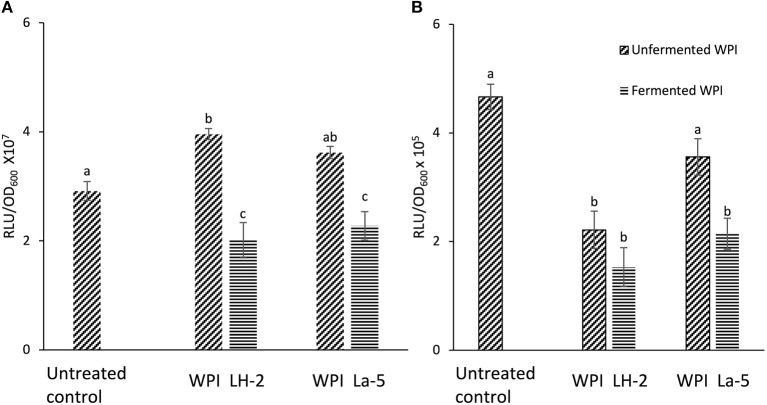
Bioluminescence of *S*. Typhimurium *hilA::lux* **(A)** and *ssrB::lux* **(B)** measured after 3 h of incubation in LB broth alone (Untreated control), LB broth supplemented with 10% non-fermented WPI-sugar CFSM (Unfermented WPI) and LB broth supplemented with 10% *L. helveticus* LH-2 or *L. acidophilus* La-5 CFSM (Fermented WPI). Data are the means ± the standard deviations derived from triplicate assays and statistical analysis was carried out using ANOVA followed by Tukey's *post hoc* test. Different letters indicate significant differences (*P*-value of ≤ 0.05) within each subsection of the figure.

Bioluminescent reporter strains *S*. Typhimurium *hilA::lux* and *ssrB::lux* were used for initial screening of the antivirulence effects of CFSM obtained from WPI fermented with *L*. *helveticus* LH-2 and *L. acidophilus* La-5. CFSM from both strains reduced luminescence of the *S*. Typhimurium *hilA::lux* while only La-5 could decrease *ssrB::lux* reporter luminescence; indicating down-regulation of the *hilA* and *ssrB* genes (Figures 1A,B). Both uninoculated control WPI had no downregulatory effect on *S*. Typhimurium *hilA::lux* light emission with a slight but significant increase in expression of the same gene in WPI glucose based media (Figure 1A). The uninoculated WPI sucrose based medium had no effect on *S*. Typhimurium *ssrB::lux* luminescence while WPI glucose based media showed significant down regulation of *S*. Typhimurium *ssrB::lux* luminescence (Figure 1B).

Bioluminescent reporter genes are considered a rapid tool for initial monitoring of gene expression. However, bioluminescence may be influenced by several factors, including other components of the medium. Previous observations using luminescent constructs suggest the occurrence of false positives and negatives with respect to virulence gene expression (12, 19, 31, 32). This is why an alternative method was chosen to complement the bioluminescence assay results. RT-qPCR is a highly sensitive and specific method for gene expression analysis.

### Effect of *L*. *helveticus* LH-2, *L. acidophilus* La-5 Bioactive Molecules and Mixture of Synthetic Peptides on Virulence Genes in *S*. Typhimurium DT 104 Wild Type and *S*. Typhimurium *oppA* Mutant Strains

**Figure 2:**
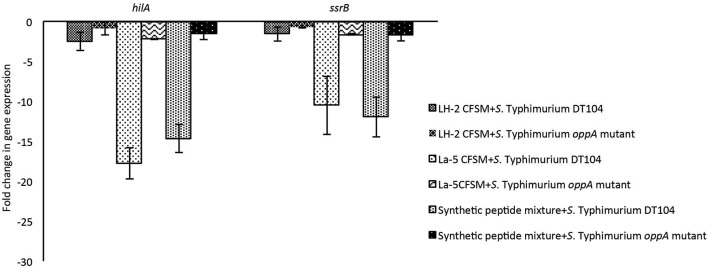
Effect of *L. helveticus* LH-2 and *L. acidophilus* La-5 CFSM on virulence gene expression of *S*. Typhimurium DT104 and *S*. Typhimurium *oppA* mutant. Expression ratios of *hilA* and *ssrB* genes of *S*. Typhimurium were normalized to the expression of the geometric average of regulator genes *16S* rRNA, *rpoD, gmk* to obtain the fold-change and compared with those of the non-fermented media.

The effects of the neutralized and reconstituted CFSM from *L*. *helveticus* LH-2 and *L. acidophilus* La-5 on virulence gene expression in multi-drug resistant *S*. Typhimurium DT104 and *S*. Typhimurium *oppA* mutant were analyzed by a 2-step RT qPCR method after incubation for 3 h (Figure 2). A statistically significant down-regulation of both the *hilA* and *ssrB* genes of *S*. Typhimurium DT 104 was observed after incubation with 10% CFSM from both LH-2 and La-5. La-5 CFSM showed stronger repression of *S*. Typhimurium DT104 *hilA* and *ssrB* genes (−17.8 and −10.49, respectively) than LH-2 CFSM (−2.56 and −1.58, respectively). The expression of *hilA* and *ssrB* genes in *S*. Typhimurium *oppA* mutant strain was −2.23 and −1.61, respectively, in the presence of La-5 CFSM while in the presence of LH-2 CFSM, expression of both genes was −0.92 and −0.77, respectively (Table 2).

**Table 2:** Effect of *L. helveticus* LH-2 and *L. acidophilus* La-5 CFSM on virulence gene expression of *S*. Typhimurium DT104 and *S*. Typhimurium *oppA* mutant compared with the control CFSM.

**Genes**	**Target strain**	**PCR efficiency**	**Cycle threshold (Ct) values** **±** **standard deviation**					
			**No treatment**	**WPI-glucose CFSM**	**LH-2 CFSM**	**WPI-sucrose CFSM**	**La-5 CFSM**	**Synthetic peptide mixture**
*16S*	*S*. Typhimurium DT104	90.25%	6.46 ± 0.45	5.65 ± 0.33	6.61 ± 0.26	5.23 ± 0.09	5.32 ± 0.08	6.27 ± 0.17
	*S*. Typhimurium *oppA* mutant		7.00 ± 0.42	6.19 ± 0.28	6.22 ± 0.34	6.21 ± 0.42	6.08 ± 0.24	6.18 ± 1.23
*rpoD*	*S*. Typhimurium DT104	93.73%	22.58 ± 0.22	21.46 ± 0.20	22.07 ± 0.09	19.37 ± 0.21	20.75 ± 0.18	23.73 ± 0.47
	*S*. Typhimurium *oppA* mutant		22.21 ± 0.18	21.22 ± 0.18	21.11 ± 0.27	21.23 ± 0.21	21.45 ± 0.24	22.86 ± 2.78
*gmk*	*S*. Typhimurium DT104	101.69%	29.19 ± 0.82	26.95 ± 0.46	28.61 ± 0.33	25.59 ± 0.58	27.96 ± 0.08	32.28 ± 0.76
	*S*. Typhimurium *oppA* mutant		31.12 ± 0.76	29.16 ± 0.54	29.27 ± 2.23	29.63 ± 0.44	29.48 ± 0.64	32.38 ± 2.76
*hilA*	*S*. Typhimurium DT104	96.37%	23.69 ± 0.36	25.31 ± 0.32	27.29 ± 0.14	20.74 ± 0.31	25.41 ± 0.17	25.94 ± 0.44
	*S*. Typhimurium *oppA* mutant		27.07 ± 0.25	27.08 ± 0.27	27.68 ± 0.48	27.74 ± 0.51	29.41 ± 0.31	30.96 ± 1.64
*ssrB2*	*S*. Typhimurium DT104	97.63%	26.29 ± 0.34	26.21 ± 0.22	27.41 ± 0.33	22.35 ± 0.06	26.67 ± 0.42	27.78 ± 0.46
	*S*. Typhimurium *oppA* mutant		26.14 ± 0.62	27.02 ± 0.26	27.05 ± 0.53	26.64 ± 0.79	27.03 ± 0.44	28.55 ± 2.41

The synthetic peptides mixture could also downregulate the expression of *hilA* and *ssrB* genes in *S*. Typhimurium DT104 (−14.66 and −11.99, respectively). The relative expression of the same genes in the *S*. Typhimurium *oppA* mutant strain in the presence of the same peptide mixture was −1.60 and −1.67, respectively (Figure 2).

### Genes Associated With Oligopeptide Metabolism in *L. helveticus* LH-2 and *L. acidophilus* LA-5

*L. helveticus* CNRZ 32 and *L. acidophilus* NCFM have previously sequenced complete genomes with the most thoroughly characterized proteolytic system (15, 33). Whole Genome Sequencing (WGS) and comparative analysis of the proteolytic components of *L. helveticus* LH-2 and *L. acidophilus* La-5 with *L. helveticus* CNRZ 32 and *L. acidophilus* NCFM, respectively, show some genetic differences in the distribution of proteolytic elements in LH-2 and La-5 (Tables 3, 4). The *L. helveticus* LH-2 genome codes for *prtH3, prtH4*, and *prtM2* CEP genes (99, 99, and 100% nucleotide identity, respectively) with the absence of *prtH, prtH2* and *prtM* genes (Table 3). The *L. acidophilus* La-5 genome codes for *prtP* and *prtM* CEP genes (99, 100% identity, respectively) (Table 4).

**Table 3:** Comparative genome analysis of the proteolytic system of *L. helveticus* LH-2 with *L. helveticus* CNRZ 32 (abbreviation LAC LHE, accession number CP002081) at the nucleotide level using NCBI microbial genome database and Blast alignment tools.

**Gene (NCBI accession number)**	**Identity (%)**
**CELL ENVELOPE-ASSOCIATED PROTEINASES**	
*prtH* (AAD50643)	0
*prtH2* (DQ826130)	0
*prtH3* (HQ602769)	4,913/4,914 (99%)
*prtH4* (HQ602770)	4,974/4,977 (99%)
*prtM* (DQ826131)	0
*prtM2* (DQ826132)	903/903 (100%)
**OLIGOPEPTIDE TRANSPORT**	
oligopeptide ABC transport protein ATP-binding component *OppF2* (AGQ24255.1)	314/314 (100%)
oligopeptide ABC transport protein ATP-binding component *OppD2* (AGQ24256.1)	351/352 (99%)
oligopeptide ABC transport protein ATP-binding component *OppF1* (AGQ23787.1)	328/328 (100%)
oligopeptide ABC transport protein ATP-binding component *OppD1* (AGQ23788.1)	342/344 (99%)
oligopeptide ABC transport protein periplasmic component *OppA4* (AGQ24260.1)	496/510 (97%)
oligopeptide ABC transport protein periplasmic component *OppA5* (AGQ24261.1)	538/540 (99%)
oligopeptide ABC transport protein ATP-binding component *OppB2* (AGQ24258.1)	309/309 (100%)
oligopeptide ABC transport protein permease component *OppC2* (AGQ24257.1) oligopeptide ABC transport protein permease component *OppC1* (AGQ23785.1)	269/269 (100%) 285/286 (99%)
**PEPTIDASES**	
Oligoendopeptidase *pepF* (AY365129)	2,363/2,370 (99%)
Oligoendopeptidase *pepO* (AF019410)	2,690/2,691 (99%)
Oligoendopeptidase *pepO2* (DQ826126)	1,939/1,947 (99%)
sialoglycoprotein endopeptidase *Ogcp* (DQ826107)	1,050/1,050 (100%)
General aminopeptidases *pepCE* (JF811429)	1,314/1,314 (100%)
General aminopeptidases *pepC* (HQ602766)	1,349/1,350 (99%)
General aminopeptidases *pepN* (U08224)	3,754/3,787 (99%)
X-prolyl diaminopeptidase *pepX* (U22900)	3,204/3,204 (100%)
Proline iminopeptidases *pepI* (DQ826125)	885/885 (100%)
Proline iminopeptidases *pepR* (U05214)	1,259/1,260 (99%)
Prolidase *pepQ* (AF012084)	2,177/2,181 (99%)
Prolidase *pepQ2* (DQ826127)	1,110/1,110 (100%)
Dipeptidase *pepD* (U34257)	2,244/2,253 (99%)
Dipeptidase *pepD2* (DQ826122)	1,422/1,422 (100%)
Dipeptidase *pepD3* (DQ826123)	1,424/1,428 (99%)
Dipeptidase *pepV* (AF012085)	2,402/2,404 (99%)
Tripeptidase *pepT* (DQ826128)	1,242/1,242 (100%)
Tripeptidase *pepT2* (DQ826129)	1,287/1,287 (100%)
Glutamyl aminopeptidase *pepA* (DQ826138)	1,083/1,083 (100%)
Methionine aminopeptidase *map* (DQ826118)	828/828 (100%)
Pyrrolidone carboxyl peptidase *pcp* (DQ826121)	603/603 (100%)
SprT-like metallopeptidase *sprT* (HQ602765)	470/471 (99%)
M16 family metallopeptidase hyp *prt2* (DQ826112)	1,257/1,257 (100%)
Membrane alanine aminopeptidase *pepM1* (HQ602768)	1,515/1,515 (100%)

**Table 4:** Comparative genome analysis of the proteolytic system of *L. acidophilus* La-5 with *L. acidophilus* NCFM (abbreviation LAC, accession code CP000033) at the nucleotide level using NCBI microbial genome database and Blast alignment tools.

**Gene (NCBI accession number)**	**Identity (%)**
**CELL ENVELOPE-ASSOCIATED PROTEINASES**	
*prtP* (LBA1512)	1,626/1,627 (99%)
*prtM* (LBA1588)	300/300 (100%)
**Oligopeptides transport**:	
Oligopeptide binding protein *oppA* (LBA0197)	1,626/1,626 (100%)
Oligopeptide ABC transporter, binding protein *oppA1B* (LBA0198)	542/542 (100%)
Oligopeptide ABC transporter, permease protein *oppB1* (LBA0200)	309/309 (100%)
Oligopeptide ABC transporter, permease protein *oppC1* (LBA0201)	343/343 (100%)
Oligopeptide ABC transporter, ATP binding protein *oppD1* (LBA0202)	352/352 (100%)
Oligopeptide ABC transporter, ATP binding protein *oppF1* (LBA0203)	313/314 (99%)
Oligopeptide ABC transporter, substrate binding protein *oppA3* (LBA1216)	527/527 (100%)
Oligopeptide ABC transporter, substrate binding protein *oppA2* (LBA1300)	584/585 (99%)
Oligopeptide ABC transporter, substrate binding protein *oppA2B* (LBA1301)	589/589 (100%)
Oligopeptide ABC transporter, permease protein *oppC2* (LBA1302)	309/309 (100%)
Oligopeptide ABC transporter, permease protein *oppB2* (LBA1303)	105/319 (33%)
Oligopeptide ABC transporter, ATP binding protein *oppF2* (LBA1305)	328/328 (100%)
Oligopeptide ABC transporter, ATP binding protein *oppD2* (LBA1306)	342/343 (99%)
Oligopeptide ABC transporter, substrate binding protein *oppA6* (LBA1400)	581/581 (100%)
Oligopeptide ABC transporter, substrate binding protein *oppA9* (LBA1961)	542/542 (100%)
**PEPTIDASES**	
Dipeptidase (LBA0035)	466/466 (100%)
Prolyl aminopeptidase (LBA0092)	293/293 (100%)
Metallopeptidase *pepO* (LBA0165)	649/650 (99%)
Pyrrolidone carboxyl peptidase (LBA0186)	200/200 (100%)
Dipeptidase *pepG* (LBA0195)	437/437 (100%)
Dipeptidase *pepE* (LBA0204)	438/438 (100%)
Prepilin peptidase (LBA0286)	228/229 (99%)
Aminopeptidase C *pepC* (LBA0343)	449/449 (100%)
Endopeptidase (LBA0390)	348/349 (99%)
Xaa-pro dipeptidase (LBA0430)	1,117/1,117 (100%)
*ampM* Methionine aminopeptidase (LBA0623)	274/275 (99%)
*pepC* Aminopeptidase (LBA0911)	437/437 (100%)
*pepD* Metallopeptidase (LBA0994)	467/467 (100%)
Signal peptidase (LBA1182)	256/257 (99%)
*pepT* Tripeptide aminopeptidase (LBA1190)	427/427 (100%)
*pepO* Metallopeptidase (LBA1275)	649/650 (99%)
*pepP* Xaa-Pro aminopeptidase (LBA1336)	1,124/1,124 (100%)
*pepQ* Metallopeptidase (LBA1343)	596/596 (100%)
*pepX* X-prolyl dipeptidyl aminopeptidase (LBA1373)	793/793 (100%)
*pepT* Tripeptide aminopeptidase (LBA1515)	427/427 (100%)
*vanY* d-alanyl-d-alanine carboxypeptidase (LBA1603)	1,312/1,312 (100%)
Prolyl aminopeptidase (LBA1658)	928/928 (100%)
*pepF* Oligoendopeptidase (LBA1763)	598/598 (100%)
*pepN* Membrane alanyl aminopeptidase (LBA1849)	844/844 (100%)
Signal peptidase (LBA1909)	210/210 (100%)
*pepL* Leucyl aminopeptidase (LBA1957)	299/299 (100%)

Comparative genome analysis of *L. helveticus* LH-2 for oligopeptide transport elements and peptidases shows the presence of these proteolytic components with high identity (97–100%) (Table 3). The *L. acidophilus* La-5 genome codes for oligopeptide transport elements and peptidases of *L. acidophilus* NCFM with high identity (99–100%), except for *oppB2* (33% identity) (Table 4).

### CEP Activities of *L*. *helveticus* LH-2 and *L. acidophilus* LA-5 in WPI

The CEP activities of intact cells from the medium fermented by *L*. *helveticus* LH-2 and *L. acidophilus* La-5 were measured based on the *in vitro* hydrolysis of the chromogenic substrate N-succinyl-Ala-Ala-Pro-Phe-p-nitroanilide. The optimal temperature for the proteinase activity of LH-2 strain was 45°C with enzyme activity 3.60 while the maximum CEP activity of La-5 was 0.25 ± 0.01 at 40°C (Table 5).

**Table 5:** Specific proteinase and aminopeptidase activities of intact cells and intracellular enzymatic extract of *L. helveticus* LH-2 and *L. acidophilus* La-5 after growth anaerobically in WPI—sugar based media at 37°C for 24 h.

**Temperature** ** (°C)**	**Specific proteinase activity**		**Specific aminopeptidase activity**	
	***L. helveticus* LH-2**	***L. acidophilus* La-5**	***L. helveticus* LH-2**	***L. acidophilus* La-5**
35	1.66 ± 0.04	0.05±*nd*	19.74 ± 0.27	5.19 ± 0.55
40	2.12 ± 0.11	0.25 ± 0.01	22.06 ± 0.60	4.08 ± 0.74
45	3.60±*nd*	0.14 ± 0.01	7.82 ± 0.91	4.43 ± 0.37
50	3.44 ± 0.03	0.21 ± 0.01	3.41 ± 0.23	2.15 ± 0.72
55	3.01 ± 0.03	0.06 ± 0.01	14.78 ± 1.32	2.75 ± 0.36

*Values are the means and SD of independent triplicates. non- detectable (nd) = SD <0·01. Specific enzyme activity is expressed as nmoles ρNa released /mg protein/min*.

### Intracellular Aminopeptidase Activities of *L*. *helveticus* LH-2 and *L. acidophilus* LA-5 in WPI

For LH-2 intracellular extract, the highest aminopeptidase activity was 22.06 ± 0.60 at 40°C while La-5 optimal activity of aminopeptidase was 5.19 ± 0.55 at 35°C (Table 5). Negligible peptidase activity was detected in the extracellular supernatant using Lys-pNa, indicating that peptidase release from the cells during our experiments was below the detection limit (data not shown).

### Peptide Profiling of CSFM of Unfermented WPI and WPI Fermented With *L. helveticus* LH-2 and *L. acidophilus* LA-5 Strains

**Figure 3:**
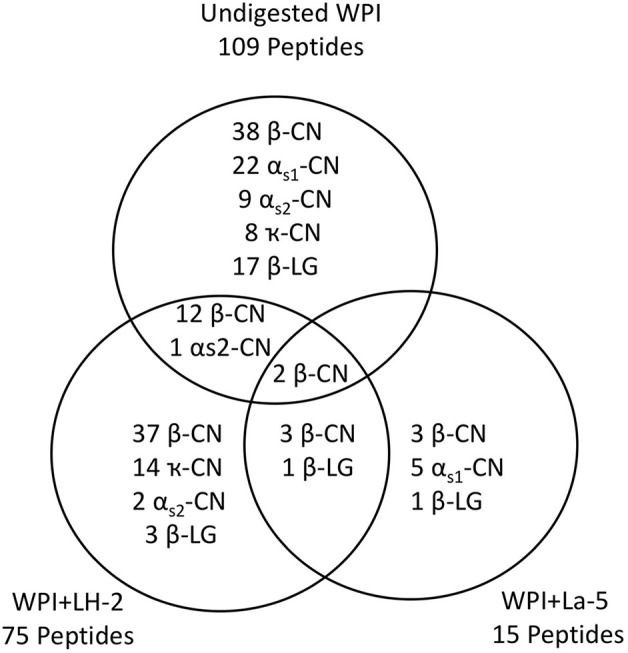
The Venn diagram shows the number and distribution of milk peptides in non-fermented WPI and WPI fermented with *L. helveticus* LH-2 or *L. acidophilus* La-5.

**Figure 4:**
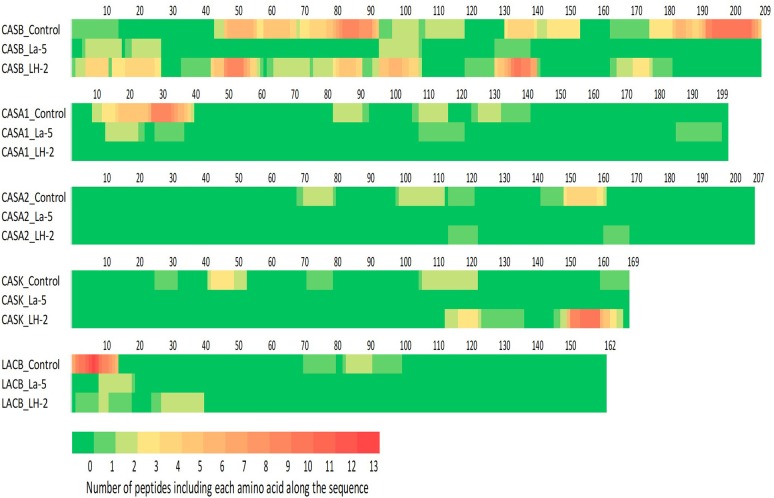
Heat maps of bovine β-, α_s1_-, α_s2_-, κ-casein, and β-lactoglobulin. Each amino acid was colored according to the number of peptides in which this amino acid was found.

**Figure 5:**
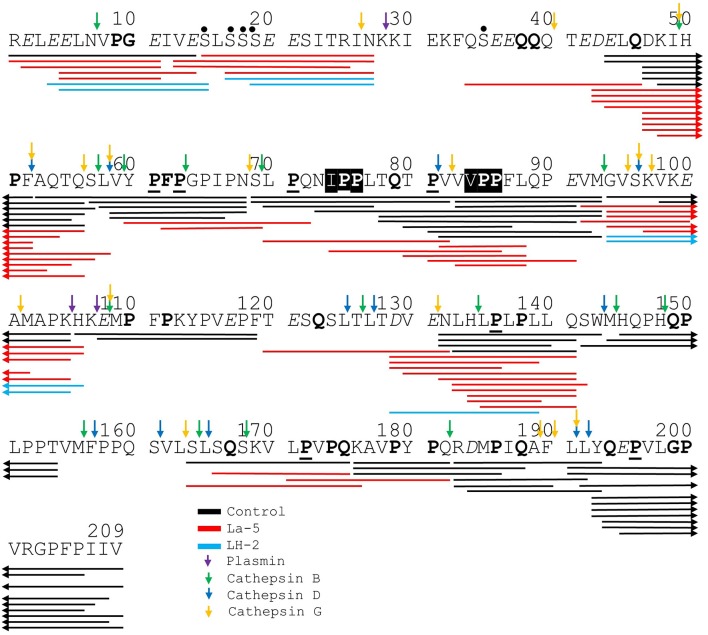
Bovine β-casein derived peptides identified in non-fermented WPI and WPI fermented with *L. helveticus* LH-2 and *L. acidophilus* La-5. Negative charged amino acids (Aspartate D, glutamate E) are in italics, Glycine G, Proline P, and Glutamine Q at position 4, 5, or 6 are marked with bold. P at second position is marked with bold and underline. VPP and IPP are inverse colors (white characters on black background). Phosphorylation sites are marked with a black dot above the serine. Cleavage sites of endogenous milk proteases are adapted from Baum et al. (34) and marked with colored vertical arrows.

**Figure 6:**
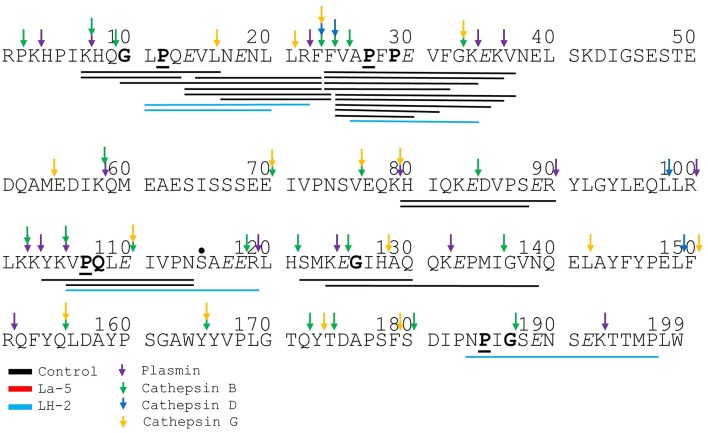
Bovine α_s1_-casein derived peptides identified in non-fermented WPI and WPI fermented with *L. helveticus* LH-2 and *L. acidophilus* La-5. Negative charged amino acids (Aspartate D, glutamate E) are in italics, Glycine G, Proline P, and Glutamine Q at position 4, 5, or 6 are marked with bold. P at second position is marked with bold and underline. VPP and IPP are inverse colors (white characters on black background). Phosphorylation sites are marked with a black dot above the serine. Cleavage sites of endogenous milk proteases are adapted from Baum et al. (34) and are marked with colored vertical arrows.

**Figure 7:**
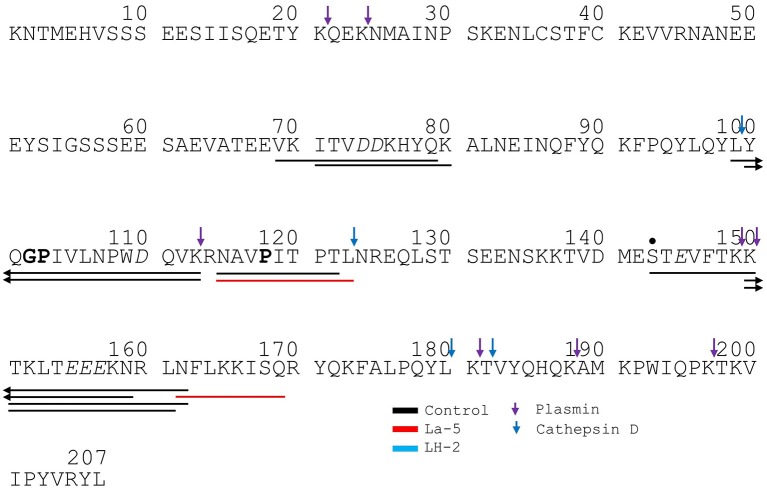
Bovine α_s2_-casein derived peptides identified in non-fermented WPI and WPI fermented with *L. helveticus* LH-2 or *L. acidophilus* La-5. Negative charged amino acids (Aspartate D, glutamate E) are in italics, Glycine G, Proline P, and Glutamine Q at position 4, 5, or 6 are marked with bold. P at second position is marked with bold and underline. VPP and IPP are inverse colors (white characters on black background). Phosphorylation sites are marked with a black dot above the serine. Cleavage sites of endogenous milk proteases are adapted from Baum et al. (34) and are marked with colored vertical arrows.

**Figure 8:**
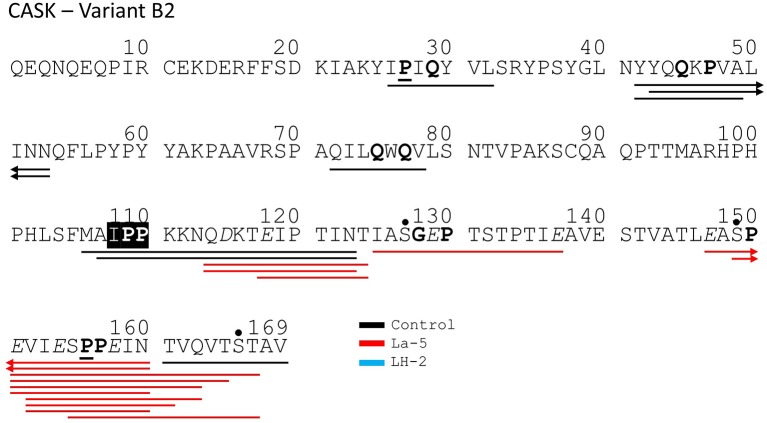
Bovine κ-casein derived peptides identified in non-fermented control WPI and WPI fermented with *L. helveticus* LH-2 or *L. acidophilus* La-5. Negative charged amino acids (Aspartate D, glutamate E) are in italics, Glycine G, Proline P, and Glutamine Q at position 4, 5, or 6 are marked with bold. P at second position is marked with bold and underline. VPP and IPP are inverse colors (white characters on black background). Phosphorylation sites are marked with a black dot above the serine. Cleavage sites of endogenous milk proteases are adapted from Hurley et al. (35) and are marked with colored vertical arrows.

**Figure 9:**
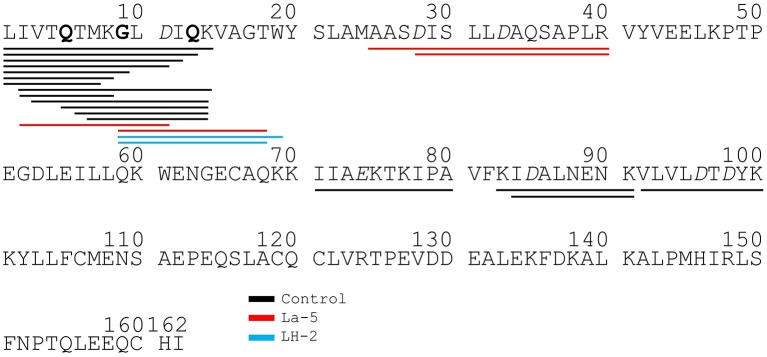
Bovine β-lactoglobulin derived peptides identified in non-fermented control WPI and WPI fermented with *L. helveticus* LH-2 or *L. acidophilus* La-5. Negative charged amino acids (Aspartate D, glutamate E) are in italics, Glycine G, Proline P, and Glutamine Q at position 4, 5, or 6 are marked with bold. P at second position is marked with bold and underline. VPP and IPP are inverse colors (white characters on black background). Phosphorylation sites are marked with a black dot above the serine.

Liquid chromatography–mass spectrometry (LC-MS) analysis of unfermented WPI and WPI fermented with LH-2 and La-5 showed that peptides are almost all fragments of the main milk proteins (109, 75, and 15 milk protein derived peptides, respectively) (Figure 3). Out of these, 56 peptides were unique to the 3-kDa fraction from LH-2 fermented medium while 9 were unique to La-5 fermented medium. CEP cleavage sites are not evenly distributed throughout the protein sequences. Regions of highest proteolytic activity are demonstrated by heat maps of peptides (Figure 4). Thus, the presence of hot spots of cleavage sites are represented by changes in color scale, where red represents more numerous peptides. The undigested WPI shows many plasmin and cathepsin cleavage sites (Figures 5–8). Endogenous milk peptides were mostly not found after fermentation with LH-2 and La-5 strains, except 14 peptides from β-casein and one peptide from α_s2_-casein (Figure 3). Most peptides found in the supernatant of LH-2 and La-5 fermented media display low-affinity residues for oligopeptide binding proteins (negatively charged amino acids, peptides with glycine, proline and glutamine at position 4, 5, or 6, peptides with proline at second position, VPP and IPP containing peptides and phosphorylated peptides) and higher resistance to peptidase hydrolysis (Figures 5–9).

Identification of bioactive peptide sequences was conducted through a search of the peptide literature databases (Table 6). Out of the 109 peptides in undigested WPI, 39 peptides have previously reported bioactivity (antihypertensive, antimicrobial, antioxidative, immunomodulatory, antithrombotic, opioid and antidiabetic activity). A total of 75 peptides were found in *L*. *helveticus* LH-2 CFSM, 19 of these peptides have previously been associated with several categories of bioactivity (ACE-inhibitory, opioid, antimicrobial and antioxidant activities). For *L. acidophilus* La-5 CFSM, a total of 15 peptides were sequenced, 5 of these peptides have previously been reported with ACE-inhibitory, antioxidant, antimicrobial and anti-caries activities. Some of these peptides have multifunctional properties that can modulate two or more physiological processes (Table 6).

**Table 6:** Bioactive peptides identified in Control WPI and WPI fermented with *L. helveticus* LH-2 and *L. acidophilus* La-5 strains by searching the Milk Bioactive Peptide Database with the reported function of the identified peptides.

**Protein**	**Peptide**	**Function**	**Control**	**LH-2**	**La-5**	**References**
Bovine β-CN	RELEELNVPGEIVE	ACE-inhibitory	X			(36)
	ELQDKIHPF	ACE-inhibitory	X			(37)
	SLVYPFPGPIPN	ACE-inhibitory	X			(38)
	LVYPFPGPIPN	ACE-inhibitory	X			(39)
	VYPFPGPI	Prolyl endopeptidase-inhibitory	X			(40)
	VYPFPGPIPN	Antioxidant, ACE-inhibitory	X			(41)
	SLPQNIPPLTQTPV	ACE-inhibitory	X			(42)
	SLPQNIPPLTQTPVVVPPFLQPEVM	ACE-inhibitory	X			(36)
	LPQNIPPL	Antidiabetic	X			(43)
	TQTPVVVPPFLQPE	Antioxidant	X			(44)
	GVSKVKEAMAPK	Antioxidant	X	X	X	(45)
	VKEAMAPK	Antibacterial,!!break Antioxidant, ACE-inhibitory	X			(45–47)
	HKEMPFPKYPVEPF	Opioid	X			(48)
	EMPFPKYPVEPF	ACE-inhibitory	X			(49)
	NLHLPLPLLQ	ACE-inhibitory	X	X		(50)
	MHQPHQPLPPT	Antirotaviral Activity	X			(51)
	HQPHQPLPPT	Immunomodulatory	X			(52)
	SLSQSKVLPVPQ	Antioxidant	X			(53)
	SQSKVLPVPQ	ACE-inhibitory	X	X		(54)
	RDMPIQAF	ACE-inhibitory	X			(42)
	LLYQEPVLGPVRGPFPIIV	ACE-inhibitory, Immunomodulatory, Antithrombin, Antimicrobial	X			(55–57)
	LYQEPVLGPVRGPFPIIV	Mitogen	X			(58)
	YQEPVLGPVRGPFP	ACE-inhibitory	X			(59)
	YQEPVLGPVRGPFPI	Antimicrobial	X			(56, 60)
	YQEPVLGPVRGPFPIIV	Immunomodulatory, Antimicrobial	X			(61)
	QEPVLGPVRGPFPIIV	ACE-inhibitory, Antioxidant	X			(59, 62)
	EPVLGPVRGPFPIIV	ACE-inhibitory	X			(45)
	DKIHPFA	ACE-inhibitory		X		(63)
	YPFPGPIHNSLPQ	Opioid		X		(64)
	LPQNIPPLTQTPV	Antidiabetic, ACE-inhibitory		X		(43, 65)
	IPPLTQTPVVVPP	ACE-inhibitory		X		(66)
	TPVVVPPFL	ACE-inhibitory		X		(37, 67)
	PVVVPPFLQPE	Antimicrobial		X		(60)
	DVENLHLPLPL	ACE-inhibitory		X	X	(68)
	LHLPLPLLQS	ACE-inhibitory		X		(69)
	HLPLPLLQ	Enteric nervous system development		X		(70)
	SLSQSKVLPVPQK	Antioxidant		X		(53)
	LPVPQKAVPYPQ	Antioxidant		X		(71)
Bovine α_s1_-CN	EVLNENLLRF	ACE-inhibitory	X			(72)
	FVAPFPEVFGK	ACE-inhibitory	X			(73)
	VAPFPEVFGKE	ACE-inhibitory	X			(74)
	VPQLEIVPNSAEER	Mineral carriers, anti-caries activity			X	(75)
Bovine α_s2_-CN	LYQGPIVLNPWDQVK	ACE-inhibitory	X			(65)
	NAVPITPT	ACE-inhibitory	X			(65)
	NAVPITPTL	Antioxidative		X		(76)
Bovine κ-CN	YYQQKPVA	Antibacterial	X			(77)
	MAIPPKKNQDKTEIPTIN	Antithrombotic, Antibacterial	X			(78)
	TVQVTSTAV	Antibacterial	X			(77, 78)
	EIPTINT	Antibacterial		X		(77)
	EVIESPPEINTVQVT	ACE-inhibitory, zinc-chelation		X		(79)
Bovine β-LG	IIAEKTKIPA	ACE-inhibitory	X			(80)
	LIVTQTMK	Cytotoxic	X			(81)
	IDALNENK	Antibacterial	X			(82)
	VLVLDTDYK	Antibacterial, DPP-IV inhibitory	X			(60, 83)
	AASDISLLDAQSAPLR	Antibacterial		X		(84)
	GLDIQKVAGT	ACE-inhibitory, Antibacterial		X	X	(60, 85)
	GLDIQKVAGTW	ACE-inhibitory			X	(85)

## Discussion

Several previous studies showed the ability of lactic acid bacteria to down regulate the expression of virulence genes of enteropathogenic bacteria (31, 86, 87). In this study, CFSM collected from WPI fermented by *L*. *helveticus* LH-2 and *L. acidophilus* La-5 reduced the expression of both the *hilA* and *ssrB* genes of the *S*. Typhimurium DT 104 wild strain. *L. acidophilus* La-5 has the most significant down-regulatory effect on the virulence genes, which indicates that the antivirulence effect is strain dependent and affected by the nature and components of CFSM. WPI fermented by La-5 contains 9 unique peptides not found in LH-2-fermented or unfermented medium.

Delcenserie et al. (32) reported that glucose could up or down-regulate the expression of virulence genes in *E. coli* O157:H7. In our study, glucose and sucrose may also affect virulence gene expression in *S*. Typhimurium. A peptidic fraction, isolated from milk fermented with *L. helveticus*, down-regulated *ssrB* gene expression of *S*. Typhimurium in the absence of glucose in the growth medium (10). CFSM from MRS without glucose fermented with some bifidobacteria species showed down-regulation of genes *hilA, ssrB2*, and *sopD* (88). These studies suggest that down-regulation of virulence genes could be caused by other non-carbohydrate metabolites produced after fermentation.

Absence of the downregulatory effect of *L*. *helveticus* LH-2 and *L. acidophilus* La-5 CFSM on the *Salmonella oppA* mutant strain signifies the importance of peptide transport inside *S*. Typhimurium to inhibit virulence gene expression. Regulation of virulence gene expression requires sensing of a specific signal in the environment such as autoinducers for quorum sensing (QS) and these signals affect the growth, metabolism and virulence of bacteria (89). Exogenous leucine increases the transport of peptides by the Opp system and enhances OppA synthesis (90). Also, activity of Opp increases the intracellular amino acid pool, which in turn activates global regulators such as Lrp or CodY (91). Baek et al. (92) reported that Lrp responds to the nutritional environment and has a repressor function on key virulence regulator genes of *S. enterica* Serovar Typhimurium by strong interaction with *hilA* and other SPI-1 and SPI-2 genes, affecting their expression by binding directly to their promoter regions P_*hilA*_, P_*invF*_ and P_*ssrA*_. From these studies and our data, it can be concluded that the presence of the *S*. Typhimurium *oppA* gene is a key factor in sensing oligopeptides, which can act as a specific signal for down regulation of virulence genes once transported into the cell.

The downregulatory effect of the synthetic peptide mixture demonstrates the antivirulence effect of specific peptides produced by *L*. *helveticus* LH-2 and *L. acidophilus* La-5 CFSM on the *S*. Typhimurium virulence genes. Also, the absence of this downregulatory effect for the *S*. Typhimurium *oppA* mutant strain shows the importance of internalization of such peptides inside *S*. Typhimurium to exhibit the antivirulence effect.

Presence of more than one CEP paralog is common among *L*. *helveticus* strains (93). Also, the presence of *prtH3* and *prtH4* with the absence of *prtH* in *L*. *helveticus* LH-2 is not unusual as *prtH* is not broadly distributed within *L. helveticus* and has no effect on growth rate in milk and cheese whey (94). *prtH* and *prtH2* distribution is also strain dependent (95). The *prtH3/prtH4* combination is common in *L. helveticus* strains (96). These studies agree with our findings for the presence of *prtH3* and *prtH4* genes and absence of *prtH* and *prtH2* genes in the genome of the LH-2 strain. *PrtM* is required for activation of *PrtH* and *PrtM2* plays a role in activation of other CEP paralogs in *L. helveticus* (97). Thus, absence of *PrtM* in LH-2 is expected as this gene was almost exclusively restricted to strains that also contained *prtH* (33).

Presence of both *PrtP* (proteinase precursor) and *PrtM* (maturase) in *L. acidophilus* La-5 confirmed that La-5 can digest large proteins extracellularly and produce small peptides. Genay et al. (95) reported that the genetic biodiversity of CEP paralogs between LAB strains could affect growth rate, strain functionality and bioactive peptide production in dairy products. This could cause differences in the antivirulence activities and bioactive peptides between of LH-2 and La-5 after growth in WPI.

Peptide transport systems can be specific for oligopeptides, or di- and tri-peptides (98). *L. helveticus* LH-2 contains all oligopeptide ABC transport elements, which is not different from previous studies of *L. helveticus* strains (99). *L. acidophilus* La-5 shows low identity of *oppB2* which is encoded by the *opp*2-type operon (100). Azcarate-Peril et al. (101) reported that Opp1 and Opp2 might have different specificities, which could explain accumulation of some peptides in La-5 growth media. Doeven et al. (98) also concluded that the lack of some elements of the membrane complex, OppBCDF, leads to presence of some peptides outside the cells due to the specificity of the transport process.

The functionality of these enzymes after genome analysis of *L*. *helveticus* LH-2 and *L. acidophilus* La-5 strains was determined by measuring the proteolytic enzyme activities. The optimal temperature for the proteinase activity of LH-2 (45°C) is not different from other previously reported *L*. *helveticus* strains (40–50°C) (102–104). The maximum CEP activity of La-5 (40 and 50°C) also is not significantly different from other *L. acidophilus* strains (45–50°C) previously reported (105).

*L*. *helveticus* LH2 CEP activity in WPI was lower than the CEP activity of other *L*. *helveticus* strains previously reported in skim milk (104, 106). The CEP activity of La-5 was slightly lower than the CEP activity of the same strain in skim milk (106) and significantly lower than the CEP activities of other *L. acidophilus* strains in chemically defined medium (107). The presence of peptides in the growth medium could inhibit the proteinase activity of some LAB (108). Mass spectrometry analysis of non-fermented WPI in this study confirmed the presence of endogenous milk protein derived peptides (β-, α_s1_-, α_s2_-, κ-casein, and β-lactoglobulin) after ultrafiltration of milk during the WPI production process. The presence of these endogenous peptides in the growth media could explain the lower CEP activities of LH-2 and La-5 in WPI compared with other related strains in milk-based growth media. The CEP activity levels of lactobacilli depend on the strain, nature and quantity of peptides present in the growth media (96). The regulation of proteinase activity is also strain dependent (107). These factors suggest that CEP activities of LH-2 and La-5 are different in WPI than other milk-based media.

*L*. *helveticus* LH-2 aminopeptidase activity in WPI based medium was significantly lower compared with previously studied *L*. *helveticus* strains after growth in simplified chemically defined medium (SCDM) supplemented with different nitrogen sources (109). *L. acidophilus* La-5 had higher aminopeptidase activity compared with *L. acidophilus* strains mentioned in Pescuma et al. (28) when grown in a chemically defined medium (CDM). The aminopeptidase activities are not influenced by the peptide content of the medium (109), so this difference may be mainly strain dependent (110).

The presence of endogenous peptides in unfermented WPI indicates the activity of endogenous milk proteases which are mainly plasmin, elastase and cathepsin D, B, and G (111). Plasmin has little or no activity toward κ-casein and whey proteins (112), so the presence of endogenous peptides from these proteins in CFSM of *L. acidophilus* La-5 and *L. helveticus* LH-2 could be due to the action of other milk proteases such as cathepsin B, D, and G (34, 113). Dallas et al. (114) concluded that minimal proteolysis by native milk enzymes continued to function during incubation in the heat-treated milk when compared with that carried out by the proteases of kefir microorganisms which were mainly *L. acidophilus* and *L. helveticus*. This observation could explain the presence of some peptides shared between the unfermented and fermented WPI. Absence of some peptides after WPI fermentation is likely due to either further hydrolysis by LH-2 and La-5 extracellular proteases or their uptake by these microorganisms. The presence of four shared peptides (three from β-casein and one from β-lactoglobulin) in WPI fermented by LH-2 and La-5 strains indicates some similarity in the affinity of the proteinases of these strains for certain cleavage sites.

According to peptide analysis data, most of the identified peptides are casein derived, even though whey was used. The presence of casein derived peptides indicates that these proteins were exposed to proteolytic cleavage during processing (115). The three dimensional structure of caseins is more open and flexible than the globular, rigid, compact structures of whey proteins, which make casein proteins more susceptible to the effect of proteases (116).Whey proteins also are not an important peptide precursor during initial fermentation or endogenous proteolysis (117). This could explain why most peptides in the unfermented and fermented WPI media originate from casein proteins.

Although β-casein is not the most abundant protein in casein, our data showed that about half (47, 72, and 53%) of identified peptides in the WPI and WPI fermented with LH-2 and La-5, respectively, were derived from β-casein. Thus, it can be concluded that the endogenous and microbial proteases of LH-2 and La-5 may preferentially attack β-casein. These findings are consistent with previously studies on bovine casein and kefir (49). The degradation of αs-casein is strain dependent (107). Our results confirm this finding, as La-5 shows different cleavage sites for αs1-casein while αs1-casein derived peptides were not found after fermentation with LH-2. Nielsen et al. (67) reported no peptides from αs1-casein digested with *L. helveticus* 1198 while Sadat-Mekmene et al. (118) concluded that αs1-casein hydrolysis was enhanced in the presence of both CEPs (*prtH, prtH2*). The lack of the *prtH* CEP gene in the *L*. *helveticus* LH-2 genome does not explain the absence of αs1-casein derived peptides in LH-2 CFSM, as some of these are present in the control medium. Strain LHC2, which has a similar combination of CEP homologs (PrtH3/PrtH4), does produce peptides from αs1-casein within a 3-h time scale (119). The longer 48-h time scale of the fermentation of WPI by LH-2 may explain the disappearance of αs1-casein peptides, and the lack of larger casein proteins in the WPI medium would preclude the release of additional αs1-casein peptides by LH-2. In contrast, LH-2 can produce αs2-casein derived peptides while La-5 cannot produce them. Pescuma et al. (107) reported that *L. acidophilus* strain CRL 636 was unable to degrade αs2-casein, which supports the current findings. LH-2 shares some κ-casein cleavage sites with other *L*. *helveticus* strains (120) while κ-casein is poorly degraded by La-5 (105). β-lactoglobulin is one of the major milk allergens (121). LH-2 and La-5 strains were able to degrade this protein, which indicates the potential of these strains in the production of hypoallergenic dairy products.

Fira et al. (105) reported that the optimal pH for CEP of *L. acidophilus* strains tested was 6.5 with a temperature optimum of 45–50°C, which might explain the lower number or diversity of peptides produced by La-5 at a lower pH and temperature (pH 4.5–5.2 at 37°C). The La-5 genome codes for only one CEP when compared with LH-2, which could result in a lower number or diversity of released peptides accumulating in the growth medium. Even though La-5 produces fewer types of peptides, those that are produced have higher antivirulence activity compare to those produced by LH-2, which depends on the nature and structure of the peptides. None of the peptides found in WPI fermented by La-5 are similar to those found in previous studies of the same strain grown in skim milk (52), which emphasizes the significant effect of growth media on the proteolytic activity of LAB.

Accumulation of peptides with predicted antivirulence effect after fermentation despite the active proteolytic and peptidolytic enzymes of *L*. *helveticus* LH-2 and *L. acidophilus* La-5 may be due to the nature and structure of these peptides. Detmers et al. (122) reported that peptides with hydrophobic and aromatic residues have high binding affinity to OppA, while those with proline, glycine, negatively charged amino acids (Aspartate D, glutamate E) and peptides with neutral or positively charged N-terminal residues having Gly, Pro and/or Gln in position 4, 5 and/or 6 significantly lower the affinity of the peptide for OppA protein. Proline at the second position of a nonameric peptide also resulted in a dramatic drop of the OppA binding affinity (122). Phosphorylated peptides and peptides high in proline content are more resistant to hydrolysis by proteolytic enzymes (123, 124). Oligopeptides containing VPP and IPP sequences could release branched chain amino acids (BCAAs) in the presence of specific peptidases. BCAAs could enhance binding of the Branched Chain Amino Acids Responsive Transcriptional Regulator (BCARR) to DNA sequences in the upstream region of the *pepV* gene, which would repress the expression of some peptidase genes in *L. helveticus* (125). Most LH-2 and La-5 peptides found in the supernatant display some of these characteristics, which may help explain their accumulation in the fermented WPI medium.

## Conclusions

*L*. *helveticus* LH-2 and *L. acidophilus* La-5 produce peptides with antivirulence effect against *Salmonella enterica* subsp. *enterica* serovar Typhimurium after growth in whey protein isolate medium. Accumulation of peptides with antivirulence activities may be related to the composition of these peptides and low affinity of these peptides to the oligopeptide-binding protein (OppA) of these strains, thus remaining in the spent medium if they are not transported into the cell. In *Salmonella*, the antivirulence activity of milk protein-derived peptides is related to the presence of the *oppA* gene. The undigested and fermented WPI by LH-2 and La-5 strains could be considered as a possible source of natural and functional ingredients, which may be used to increase the biological activity of food products. Further studies are required to explore the antivirulence ability of individual synthetic peptides with the same sequence at different concentrations compared to the antivirulence activity of fermented WPI.

## Data Availability Statement

The datasets generated for this study can be found in the the University of Guelph Research Data Repository [https://doi.org/10.5683/SP2/43M6GX].

## Author Contributions

EA carried out the experiments, interpreted the results, and wrote the first draft of the manuscript. SN participated in interpretation and presentation of the peptide results. SA-E, AE-L, and ES provided help for interpretation of the results. GL was involved in the design of the study, interpretation of the results, and writing of the manuscript.

### Conflict of Interest

The authors declare that the research was conducted in the absence of any commercial or financial relationships that could be construed as a potential conflict of interest.

## Supplementary Material

Supplementary Table S1 Information used to identify peptides in unfermented control medium and media fermented by strain La-5 or LH-2.
